# Analytical Validation of NavDx, a cfDNA-Based Fragmentomic Profiling Assay for HPV-Driven Cancers

**DOI:** 10.3390/diagnostics13040725

**Published:** 2023-02-14

**Authors:** Alicia Gunning, Sunil Kumar, Cassin Kimmel Williams, Barry M. Berger, Stephen P. Naber, Piyush B. Gupta, Catherine Del Vecchio Fitz, Charlotte Kuperwasser

**Affiliations:** Naveris, Waltham, MA 02451, USA

**Keywords:** NavDx, HPV, oropharyngeal, cancer, papillomavirus, profiling assay, OPSCC

## Abstract

The NavDx^®^ blood test analyzes tumor tissue modified viral (TTMV)-HPV DNA to provide a reliable means of detecting and monitoring HPV-driven cancers. The test has been clinically validated in a large number of independent studies and has been integrated into clinical practice by over 1000 healthcare providers at over 400 medical sites in the US. This Clinical Laboratory Improvement Amendments (CLIA), high complexity laboratory developed test, has also been accredited by the College of American Pathologists (CAP) and the New York State Department of Health. Here, we report a detailed analytical validation of the NavDx assay, including sample stability, specificity as measured by limits of blank (LOBs), and sensitivity illustrated via limits of detection and quantitation (LODs and LOQs). LOBs were 0–0.32 copies/μL, LODs were 0–1.10 copies/μL, and LOQs were <1.20–4.11 copies/μL, demonstrating the high sensitivity and specificity of data provided by NavDx. In-depth evaluations including accuracy and intra- and inter-assay precision studies were shown to be well within acceptable ranges. Regression analysis revealed a high degree of correlation between expected and effective concentrations, demonstrating excellent linearity (R^2^ = 1) across a broad range of analyte concentrations. These results demonstrate that NavDx accurately and reproducibly detects circulating TTMV-HPV DNA, which has been shown to aid in the diagnosis and surveillance of HPV-driven cancers.

## 1. Introduction

Human papillomavirus (HPV) is the etiologic agent responsible for the vast majority of oropharyngeal as well as anogenital carcinomas [1]. HPV-driven oropharyngeal squamous cell carcinoma (OPSCC) has become the most common of the HPV-associated cancers in the United States, overtaking cervical cancer diagnoses in recent years as predicted [2,3]. Incidence of HPV-driven OPSCC, which results from chronic HPV infection established primarily in the tonsil and palatine lymphoid tissue, has been one of the most rapid to rise of all cancers in high-income countries [4,5,6].

Approximately 15–25% of patients with HPV-driven OPSCC will have recurrence with locoregional or distant metastatic disease within three years [7,8,9,10,11,12,13,14]. Accurate, early identification of residual and recurrent OPSCC is therefore critical to ensuring the timely initiation of additional therapy to preserve beneficial outcomes. Standard care treatment for patients with HPV-associated OPSCC includes surgical resection and/or chemo/radiotherapy [15]. Most US institutions perform a PET/CT imaging scan at 10 to 16 weeks after treatment to assess response. Typically, patients with a negative PET/CT scan are observed and may receive an additional six PET/CT scans over a five year follow-up period [16]. There is a demonstrable unmet need for an accessible, reliable surveillance test with low patient and clinical practice site impact that could provide an early indication of recurrent HPV-driven OPSCC. A broadly accessible blood test could meet this need.

Circulating tumor DNA released by cancer cells represents a source of OPSCC tumor genomic biomarkers accessible by simple phlebotomy [17,18]. Often referred to as liquid biopsy assays, circulating cell-free DNA-based assays are a growing method for providing genomic profiling results of patients [19,20]. Blood-based assays could provide a less invasive, more sensitive and specific diagnostic method to detect the presence of cancer, or quantify tumor burden among patients with HPV-associated tumors [21,22,23,24,25,26,27,28,29,30,31,32,33]. Patients with HPV-driven malignancies are “infected with HPV” by definition. Conventional DNA testing for HPV, however, does not distinguish HPV DNA attributed to active or chronic viral infection alone from tumor-associated HPV DNA. Naveris has therefore developed NavDx, a clinically validated blood-based assay to specifically detect circulating tumor tissue modified HPV (TTMV-HPV) DNA arising from HPV-driven malignancies [34,35,36,37,38,39,40,41,42].

We validated the NavDx assay based on the Clinical Laboratory Improvement Amendments (CLIA ‘88) regulations for laboratory-developed tests in clinical chemistry and the matching Clinical and Laboratory Standards Institute guidelines. This included the evaluation of limit of the blank (LOB), limit of detection (LOD), limit of quantification (LOQ), stability, accuracy, intra-assay and inter-assay precision, and dilution linearity. Here, we present the analyses of the broad analytical validation of NavDx.

## 2. Materials and Methods

### 2.1. Test Characteristics

The NavDx assay is a CLIA high complexity laboratory developed test used as an aid in the detection of human papillomavirus (HPV)-driven cancer. NavDx, which utilizes circulating cell-free DNA isolated from plasma derived from the anti-coagulated peripheral whole blood of cancer patients, was developed, validated, and performed by Naveris, Inc (Waltham, MA) under CLIA regulations. Naveris’ laboratory is accredited by the College of American Pathologists and the New York Department of Health Wadsworth Center, and the validation included in this publication was available and reviewed by onsite inspectors. NavDx uses 12 DNA biomarkers to detect and profile the fragmentation pattern of HPV DNA using droplet digital PCR (ddPCR). This novel methodology for fragment size assessment provides a superior limit of detection for tumor-derived HPV DNA compared to standard digital PCR assays for HPV genes E6 and E7. Importantly, this approach allows NavDx to discriminate between HPV DNA from malignant and non-malignant sources [34,35,36,37,38,39,40,41,42]. A quantitative algorithm differentially weights the circulating HPV DNA fragments based on size to generate a TTMV-HPV DNA prognostic risk score that reflects the quantity of circulating tumor derived HPV DNA. All 5 high-risk HPV types associated with OPSCC (16, 18, 31, 33, 35) are identified and reported. The detection of an internal control gene, *ESR1*, is used to assess the quality of DNA from different specimens.

### 2.2. Bioinformatics

After plasma is separated from peripheral blood, the DNA is isolated and analyzed by ddPCR using 12 probes and 24 primers targeting genomic regions in 5 high-risk HPV strains. Another probe with 2 primers directed at *ESR1* serves as a control. Each probe and primer pair combination represents one amplicon target within an HPV genome, and the collection of probes and primers are designed to allow computational analysis of the data to determine the quantity of the viral DNA fragments detected.

For HPV18, 31, 33 and 35, ddPCR data were analyzed using QuantaSoft software version 1.7.4.0917 (Bio-Rad, Hercules, CA, USA). For HPV16, K-means analysis is performed on a reference set of aggregated data to create a set library. An individual reference set only contains aggregated data for a single type of multiplexed ddPCR reaction and combines raw multiplexed ddPCR data across over 300 clinical and analytic samples processed in the laboratory. The K-means analysis provides initial locations for a Gaussian mixture model analysis, which is fitted on the reference set to identify cluster locations for each type of multiplexed ddPCR reaction. Learning for the mixture model is performed through an iterative expectation–maximization algorithm. 

The Gaussian model is applied to each droplet in the multiplex reaction to provide a posterior probability vector, assigning probabilities that a droplet belongs to a given cluster. Droplets with probability >0.99 for a single cluster are considered uniquely assigned to that cluster. Reactions for which >98% of droplets are uniquely assigned to a single cluster pass quality control. 

The number of droplets assigned to each cluster is tabulated. In cases where droplets are not uniquely assigned to a single cluster, the posterior probability weights previously determined for the droplet are used in the tabulation. Counts for each of these clusters are summed in a weighted linear combination and regression normalized to generate the TTMV-HPV DNA score. 

### 2.3. Validation Materials

With regard to the stability assessment, specimens were submitted to the commercial/CLIA laboratory at Naveris for routine NavDx testing. Institutional Review Board (IRB) approval was not required as this study falls under the Health Insurance Portability and Accountability Act (HIPAA) safe harbor for quality assurance activities within the laboratory performing the assay.

Engineered samples were used for reference interval studies. We diluted plasmid purified HPV DNA, encoding for whole plasmid and/or synthesized target-specific DNA regions of HPV16, HPV18, HPV31, HPV33 and HPV35 to varying concentrations in TE buffer and/or water for all reference studies.

### 2.4. Determination of Assay Performance Characteristics

#### 2.4.1. Stability of TTMV-HPV DNA Analytes from Blood

Tests used stored plasma from peripheral blood collected into 10-mL Streck tubes (Streck, La Vista, NE, USA; catalog #230471). The samples were centrifuged at 4 °C (2000× *g*) for 10 min, followed by additional centrifugation of the separated plasma at 4 °C (2500× *g*) for 10 min to create platelet-poor plasma. cfDNA was extracted from 4 mL of platelet-poor plasma with the Qiagen Circulating Nucleic Acid kit following the manufacturer’s protocol. We analyzed extracted DNA from plasma samples at days 1 through 14 after storage at ambient temperature for a housekeeping gene (*ESR1*). Patient samples were sorted by the length of time, in days, between local blood collection and extraction at the Naveris Laboratory in Natick, MA, USA. DNA stability was evaluated daily for days 1–7 and as a group for specimens stored from 8 to 14 days. DNA recovery was reported as the average as well as the minimum and maximum detected *ESR1* per mL of plasma isolated. Per Naveris protocols, samples were rejected if the *ESR1* values were less than 500 fragments/mL plasma. The TTMV-HPV DNA percent positivity rate was reported daily for days 1–7, for the group for days 8–14, and for the entire sample population. All methods were in alignment with current good laboratory practice and CLIA guidelines.

#### 2.4.2. Specificity (Limit of Blank)

Specificity was measured by determining the Limit of Blank (LOB) as defined according to CLSI guidelines adapted to ddPCR. The LOB is the highest quantity value that is likely to be observed, with a stated probability, for a blank material (i.e., zero copies of the analyte under measure). Specificity or LOB was determined by analyzing blanks, or No Template Controls (NTC), consisting of molecular grade water, with no DNA. A total of 18 samples per type were run (six samples on three separate days) to ascertain the LOB for the TTMV-HPV DNA analytes.

#### 2.4.3. Sensitivity (Detection Limit)

To determine the Detection Limit or Limit of Detection (LOD), sequence verified HPV Target Sequence DNA was used to measure the absolute quantification of TTMV-HPV16, 18, 31, 33, and 35 DNA by digital droplet PCR (ddPCR). Target sequence solutions were prepared for each analyte, and three replicates of six titration standards (40, 20, 10, 5, 3 and 1 copies/μL) were used to determine the limit of quantitation for each assay. The Limit of Quantitation (LOQ) is the level at which the analyte can be measured in less than 80% of the samples. The LOD is the lowest analyte concentration likely to be reliably distinguished from the LOB and at which detection has an RSD < 20%. Both LOQ and LOD were defined according to CLSI guidelines adapted to ddPCR. The LOD was determined by utilizing both the measured LOB and the concentration of analyte at the LOQ, and calculated using the formula:

LOD = LOB + 1.645(SD_LOQ concentration sample_).

#### 2.4.4. Accuracy, Precision and Linearity

We determined analytical accuracy, intra-assay and inter-assay precision, and linearity for each HPV type using six different concentrations of engineered HPV plasmid DNA. Samples were prepared by diluting the HPV Target Sequence to a nominal sample preparation concentration. Three replicate sample solutions for HPV16, HPV18, HPV31, HPV33, and HPV35 were prepared with final ranges of concentrations between 2000 copies/µL and 8 copies/µL for all assays (see Appendix A). Analytical accuracy was assessed by measuring analyte concentration (copies/μL) in samples with known levels of analytes and reported as percent recovery of the known concentration or as the difference between the mean and the accepted true value. Intra-assay precision (repeatability or method precision) evaluates the variation experienced within a run by a single technologist on a single instrument. Inter-assay (or intermediate) precision refers to variations within-lab on different days with different instruments. Each of the control sample solutions was tested in triplicate at six different concentrations for intra-assay precision, and at six different concentrations on three different days, as well, for inter-assay precision. Samples were analyzed and coefficients of variation (% CV) for the mean effective concentrations were calculated in both studies. Linearity evaluates the ability within a given range to obtain a response that is directly proportional to the concentration of analyte standard. To measure the linearity of NavDx, six standard solutions of analyte for each type were prepared as described in methods for accuracy and precision. Samples were analyzed and linear regression analysis was performed for analyte concentration versus signal response (or effective concentration) of analyte.

## 3. Results

### 3.1. Stability of TTMV-HPV DNA Analytes in Blood

The Streck BCT manufacturer’s specifications for circulating tumor cell (CTC) sample stability are seven days at 15 °C to 30 °C, and the specifications for circulating cell-free DNA (cfDNA) and genomic DNA (gDNA) sample stability are 14 days at 6 °C to 37 °C [43]. Additional corroboration of the seven-day stability of the NavDx assay was examined by reviewing the housekeeping gene *ESR1* as well as TTMV-HPV DNA positivity on days one through seven, the acceptable interval for specimen acceptability for NavDx testing. Percent positivity for TTMV-HPV DNA remained steadily at 26.2–32.5% on days one through seven. The specimen integrity for samples received at days eight to 14 was also examined. Results pooled from days eight to 14 displayed a 29.7% positivity rate and marginal increase in *ESR1* values due to an increase in cellular DNA release with time [44]. Results can be seen in Table 1. While the NavDx test was shown to be able to recover acceptable concentrations of DNA and TTMV-HPV DNA analytes from blood beyond seven days post sample collection, a seven-day cut off for stability was chosen to ensure the enhanced specificity of NavDx. 

### 3.2. Detection Capability

#### 3.2.1. Specificity (Limit of Blank)

Of the 18 NTD samples for TTMV-HPV16 DNA detection, eight were completely blank and ten showed weak background positivity ranging from 0.23 to 0.83 copies/μL. Based on these results, the LOB for the TTMV-16 assay averages to 0.32 copies/μL. Looking at the 18 NTD samples for TTMV-HPV18/33 DNA run over three separate days, thirteen were completely blank and five NTD samples showed weak positivity ranging from 0.20 to 0.23 copies/μL. As such, the LOB for the TTMV-HPV18/33 assay FAM channel is 0.15 copies/μL (HPV33) and for the HEX channel is 0.17 copies/μL (HPV18). For TTMV-HPV31/35 DNA, 14 of the 18 NTD samples analyzed were completely blank while four NTD samples showed weak positivity at a concentration of 0.20 copies/μL. Based on these results, the LOB for the TTMV-31/35 assay FAM channel is 0.19 copies/μL (HPV35) and for the HEX channel it is zero copies/μL (HPV31). A list of LOBs can be found in Table 2.

#### 3.2.2. Sensitivity (Detection Limit)

The Limit of Quantitation (LOQ) was determined for each HPV type using titration samples from 40 down to one copies/μL. LOQs for TTMV-HPV16, 18, 31, 33 and 35 DNA are as follows: <1.20, 3.56, 4.11, 4.00, and 3.50 copies/μL. The Limit of Detection (LOD) for each corresponding type was calculated utilizing both the measured LOB and the concentration of analyte at the LOQ. LODs for TTMV-HPV16, 18, 31, 33 and 35 DNA are as follows: 0.56, 1.31, 0.63, 1.10 and 0.57 copies per microliter (copies/μL). Results for LOQs and LODs are presented in Table 2.

### 3.3. Analytical Accuracy

Prepared HPV samples for all five types (TTMV-HPV16, 18, 31, 33, 35) consisted of six concentrations ranging from 2000 down to eight copies per μL (see Appendix A). All individual percent recoveries for every sample tested were measured and calculated between 83.8% and 130%, while mean percent recoveries for all types tested fell between 92.7% and 112.6%. See Table 3 for a TTMV-HPV DNA type specific breakdown of mean percent recoveries reflecting analytical accuracy.

### 3.4. Precision Studies

Intra-assay or method precision was determined by measuring the effective concentrations for six concentrations for each TTMV-HPV DNA type. Relative standard deviations ranged from 2.1 to 13.0%, 2.1 to 13.7%, 1.0 to 9.0%, 1.5 to 13.9% and 1.1 to 7.1% for TTMV-HPV16, 18, 31, 33 and 35 DNA, respectively. Variations within the Naveris laboratory on different days were assessed and the recovery values were calculated and reported as a means to determine inter-assay or intermediate precision. A statistical comparison is made across the days’ results. As concentrations decreased, the %CVs for all types remained either at or well under 20%, ranging from 2.9 to 9.0% for TTMV-HPV16, 2.4 to 20% for TTMV-HPV18, 1.9 to 16.5% for TTMV-HPV31, 2.5 to 13.1% for TTMV-HPV33, and 1.8 to 11.2% for TTMV-HPV35. Results from precision studies can be found in Table 4.

### 3.5. Linearity

**Figure 1:**
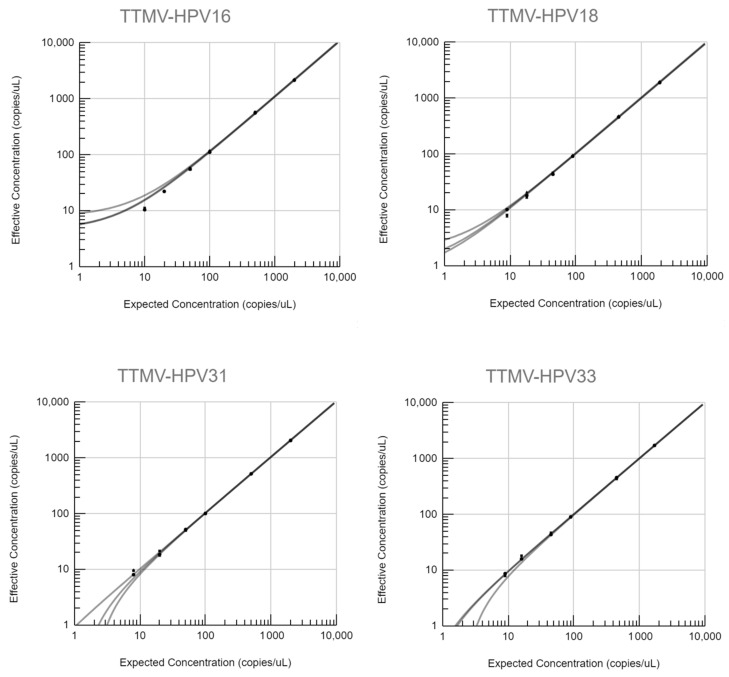

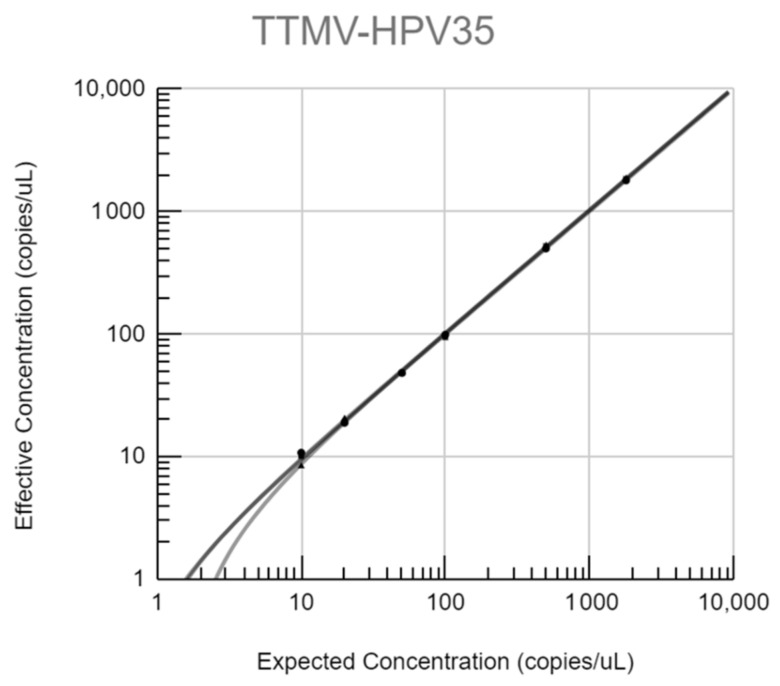
Linearity plots for TTMV-HPV16, 18, 31, 33 and 35 DNA at six different concentrations on days one, three and five. Corresponding equations and R^2^ values are given in Table 5 below.

The ability of the NavDx test to obtain a response that is directly proportional to the concentration of analyte standard was assessed. The slopes of expected-to-effective concentrations ranged from 0.997 to 1.11 and intercepts from −2.31 to 7.96. Coefficients of determination equaled one (R^2^ = 1) for all TTMV-HPV DNA types at all time points. Linear plots at time points for days one, three and five are depicted in Figure 1. Table 5 contains equations and R^2^ values for all types. Linearity (R^2^ > 0.99) can also be seen with the LOQ results on the lowest end of the range (down to 1 copy/µL; see Appendix B).

## 4. Discussion

Human papillomavirus (HPV)-driven oropharyngeal squamous cell carcinoma (OPSCC) is one of the most rapidly rising cancers today [4,5,6]. Accessible, reliable testing with low patient and clinical practice site impact is needed to provide diagnosis, early indication of recurrence, and monitoring of HPV-driven cancers. The challenge is that all patients with HPV-driven malignancy are by definition infected with HPV. Historically, however, DNA testing for HPV does not distinguish HPV DNA attributed to active or chronic viral infection alone from HPV DNA that originates from an HPV-related malignancy.

NavDx is a proprietary laboratory-developed diagnostic assay developed and validated by Naveris to specifically detect HPV DNA originating in HPV-driven malignancy, denoted herein as circulating tumor tissue modified viral (TTMV)-HPV DNA, for the monitoring of patients with HPV-driven cancers [34,35,36,37,38,39,40,41,42]. As an accessible, reliable blood test with low patient and clinical site acquisition impact, NavDx provides a significantly earlier indication of HPV-driven OPSCC recurrence than current clinical practices and is able to distinguish TTMV-HPV DNA from HPV DNA originating from infection. 

The analyses put forth in this publication do not seek to demonstrate the clinical utility of NavDx, nor do they provide a comparison to other assays that detect circulating HPV DNA. Published NavDx clinical performance characteristics in patients with recurrent HPV-associated OPSCC demonstrate positive and negative predictive values (PPV and NPV) of 97.9% and 95.7%, along with sensitivity and specificity of 90.4% and 98.6% [34,35,36,37,38,39,40,41,42] (see Appendix C). We feel that the clinical validity results referenced strongly suggest that NavDx performance would meet or exceed that of comparable assays, but that type of analysis would extend beyond the scope of this manuscript, which focuses solely on the analytical validation of the NavDx test.

The stability of patient blood samples used with the NavDx test was analyzed, demonstrating that acceptable concentrations of DNA and TTMV-HPV DNA analytes could be recovered beyond seven days post-sample collection using Streck tubes [43]. As individual patient samples cannot be examined over a 14-day time course due to sample volume limitations, large sample sizes were analyzed and percent positivity measured. A conservative seven-day cut off for acceptable specimens for clinical analysis was chosen for accepting blood samples for analysis by NavDx.

NavDx specificity and detection limits for TTMV-HPV DNA were measured by determining the Limits of Blank (LOBs), Limits of Quantitation (LOQs) and Limits of Detection (LODs) for each HPV type analyzed by the NavDx assay. The assay showed high specificity, as demonstrated by the LOBs, found to be between 0 and 0.32 copies/μL for TTMV-HPV16, 18, 31, 33 and 35. LOQs were measured using dilutions from 40 to one copies/μL and shown to be <1.20 to 4.11 copies/μL and linear for all types analyzed, with standard deviations below 20%. LODs were calculated using these data and determined to be 0.56–1.31 copies/μL for the five types, highlighting the high level of sensitivity achievable with NavDx. 

Prepared HPV target sequences for all five types (TTMV-HPV16, 18, 31, 33, 35) over a series of six dilutions were used to measure analytical accuracy, precision, linearity and range. For all five types, analytical accuracy was shown to be within ranges and precision studies revealed satisfactory method/within-run as well as intermediate/within-lab precision. As expected, when analyzing the lowest concentrations, slight variations represent higher proportions of the expected concentration and therefore %CVs are increased, while in most cases at higher concentrations the measurements were much tighter, with %CVs at 1–6%. Regression analyses demonstrated strict linearity with all five types, with coefficients of determination at one (R^2^ = 1) in every case. Linearity can be seen down to the Limit of Quantitation (see Appendix B). When TTMV-HPV DNA scores exceed the upper limit of the range of the assay (designated here as 2,000), samples are diluted pre-measurement to ensure the accuracy of the readings. In these cases, dilutions of specimens exceeding the linear range correlate well with expected levels of the original diluted analyte. Across a broad range of concentrations, NavDx has been shown to provide results directly proportional to the analyte concentration in question.

## 5. Conclusions

While clinical validation for NavDx has been well documented in the literature [34,35,36,37,38,39,40,41,42] (see Appendix C), the analytical validity of the NavDx test has not previously been presented outside of the materials provided to the CLIA and accrediting bodies. Here, we have provided analytical validation for NavDx including a thorough investigation of the stability of whole blood samples up to seven days, high specificity given the results of the low values of LOBs, and high sensitivity shown by similarly low LOQs and LODs. Analyses also showed good accuracy and precision, both intra- and inter-assay, and excellent linearity. These studies demonstrate the reliability of NavDx as a laboratory-based assay for use in detecting circulating fragments of TTMV-HPV DNA from five high risk HPV subtypes commonly found in HPV-driven cancers.

## 6. Patents

Gupta, G; Chera, BS; Kumar, S; Method for Quantifying DNA Fragments in a Sample by Size. US 11168373 B2, 2021.

Gupta, G; Chera, BS; Kumar, S; Compositions and Methods for the Selective Detection of Tumor-Derived Viral DNA. US 11254989 B2, 2022.

## Figures and Tables

**Table 1:** Stability of TTMV-HPV DNA analyte detection in blood measured by percent positivity. *ESR1* values are given in fragments per milliliter of plasma (frg/mL).

Day(s)	Average (Min-Max) *ESR1* Values (frg/mL)	#TTMV-HPV Positive Cases	Sample Size (N)	% Positivity
1	3528 (504–436,345)	981	3396	28.9
2	3578 500–843,085)	1915	7321	26.2
3	4779 (506–920,213)	675	2322	29.1
4	4224 (528–834,135)	1126	4322	26.1
5	4531 (506–433,125)	617	2115	29.2
6	7693 (623–683,446)	200	615	32.5
7	5005 (505–70,565)	48	179	26.8
8–14	7581 (675–161,538)	38	128	29.7
Totals		5600	20,398	27.5

**Table 2:** Limit of Blank (LOB), Limit of Quantitation (LOQ), and Limit of Detection (LOD) for each TTMV-HPV DNA type given in copies per microliter (copies/μL).

	Copies/μL		
Type	LOB	LOQ	LOD
TTMV-HPV16	0.32	<1.20	0.56
TTMV-HPV18	0.17	3.56	1.31
TTMV-HPV31	0	4.11	0.63
TTMV-HPV33	0.15	4.00	1.10
TTMV-HPV35	0.19	3.50	0.57

**Table 3:** Analytical accuracy expressed as mean percent recoveries for TTMV-HPV DNA types over a range of dilutions with prepared HPV samples.

	Mean % Recoveries				
Dilution *	TTMV- HPV16	TTMV- HPV18	TTMV- HPV31	TTMV- HPV33	TTMV- HPV35
1:1	107.2	99.7	102.6	101.1	99.9
1:4	111.5	101.9	103.6	101.2	110.5
1:20	110.6	101.4	100.5	100.4	98.9
1:40	110.5	96.5	104.0	96.6	96.5
1:100	110.3	101.1	92.7	99.4	94.5
1:200	104.3	112.6	100.5	96.3	107.7

* See concentrations listed in Appendix A.

**Table 4:** Intra-assay precision (repeatability) and inter-assay precision (within-lab on differing days), reflected by percent relative standard deviations of effective concentrations of the five TTMV-HPV DNA types measured at six concentrations in triplicate.

Dilution *	%CVs of Mean Effective Concentration (Copies/μL)									
	Intra-Assay					Inter-Assay				
	TTMV-HPV16	TTMV-HPV18	TTMV-HPV31	TTMV-HPV33	TTMV-HPV35	TTMV-HPV16	TTMV-HPV18	TTMV-HPV31	TTMV-HPV33	TTMV-HPV35
1:1	2.1	2.7	2.2	3.0	1.4	2.9	2.4	1.9	2.5	1.9
1:4	2.1	2.1	1.0	1.5	3.5	3.3	3.6	2.6	4.3	3.8
1:20	3.3	2.7	3.0	5.4	1.2	4.7	2.9	6.3	4.9	3.0
1:40	2.0	6.2	8.0	11.5	1.1	4.6	5.4	7.5	8.4	6.4
1:100	12.2	10.1	9.0	5.3	7.1	7.5	11.9	10.8	10.8	5.8
1:200	13.3	13.8	7.2	13.9	4.7	9.0	20.0	16.5	13.1	11.2

* See concentrations listed in Appendix A.

**Table 5:** Equations and coefficients of determination (R^2^ values) for all types at days one, three and five. Corresponding linearity plots are given in Figure 1 above.

Type	Time	Series	Equation	R^2^ Value
TTMV-HPV16	Day 1	⬤	y = 1.07x + 4.61	1
	Day 3	▲	y = 1.11x + 4.65	1
	Day 5	◾	y = 1.08x + 7.96	1
TTMV-HPV18	Day 1	⬤	y = 0.998x + 1.91	1
	Day 3	▲	y = 1.04x + 0.962	1
	Day 5	◾	y = 1.01x + 0.706	1
TTMV-HPV31	Day 1	⬤	y = 1.03x − 0.11	1
	Day 3	▲	y = 1.04x − 2.3	1
	Day 5	◾	y = 1.05x − 1.46	1
TTMV-HPV33	Day 1	⬤	y = 1.01x − 0.687	1
	Day 3	▲	y = 1.01x − 0.579	1
	Day 5	◾	y = 0.997x − 2.31	1
TTMV-HPV35	Day 1	⬤	y = 0.999x − 0.581	1
	Day 3	▲	y = 1.03x − 0.681	1
	Day 5	◾	y = 1.03x − 1.62	1

**Figure A1:**
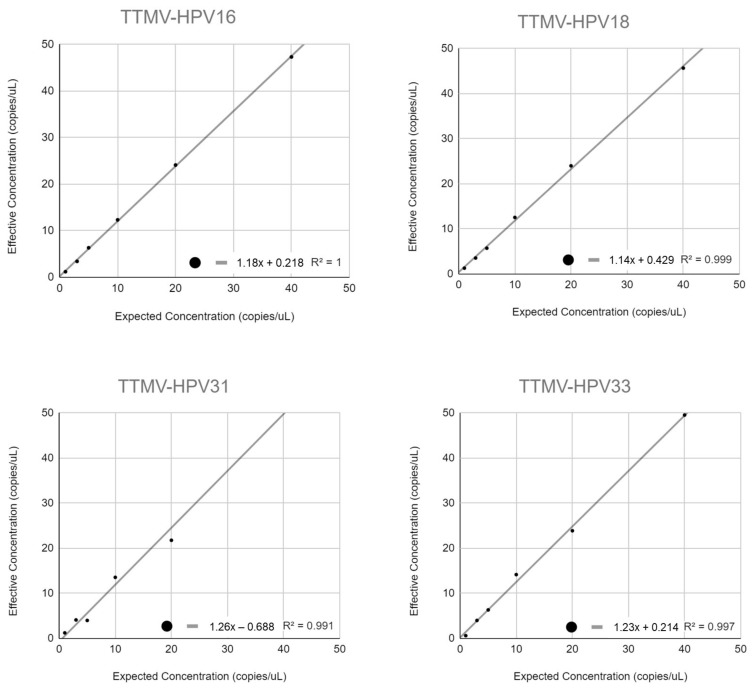

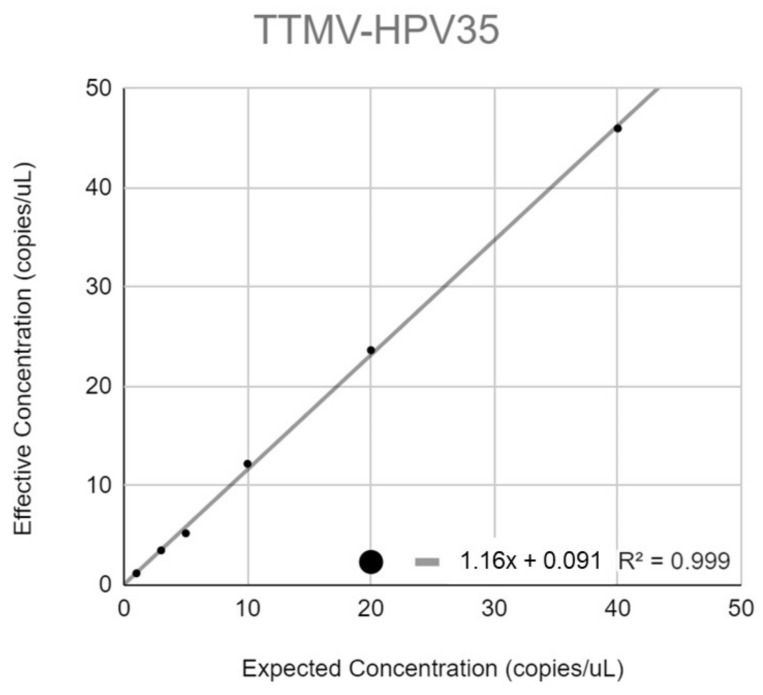
Linearity represented as expected-to-effective concentrations for TTMV-HPV16, 18, 31, 33 and 35 at 1–40 copies/µL, with respective equations and coefficients of determination.

## Data Availability

The data generated in this study are not publicly available but are available upon reasonable request from the corresponding author.

## Appendix A

**Table A1:** Dilution concentrations used in Accuracy, Precision and Linearity studies given in copies/μL.

HPV16	HPV18	HPV31	HPV33	HPV35
2000	1900	2000	1700	1800
500	450	500	450	500
100	90	100	90	100
50	45	50	45	50
20	18	20	16	20
10	9	8	9	10

## Appendix C

**Table A2:** NavDx sensitivity and specificity data from published studies evaluating the tumor tissue modified viral HPV-DNA in patients with OPSCC-driven oropharyngeal cancer, alongside control populations of patients with non-HPV-driven cancers and biobank samples from individuals not known to have HPV-related disease.

Metric	Reference	Method	Result	N	Detected	95% C.I.
**Sensitivity**	Chera, 2019 [34]	OPSCC tissue HPV+ by p16 ^1^	89.3%	103	92	
	Rettig, 2022 [38]	OPSCC tissue HPV+ by p16 and/or RNA in situ hybridization (ISH) ^2^	89.1%	110	98	
	Routman, 2022 [36]	OPSCC tissue HPV+ by p16 or RNA in situ hybridization (ISH)	88.9%	45	40	
	Chung, 2022 [39]	OPSCC tissue HPV+ by p16	94.6%	37	35	
	Echevarria, 2022 [42]	OPSCC tissue HPV+ by p16	90.9%	33	30	
	Gerndt, 2021 [41]	OPSCC tissue HPV+ by p16	93.5%	46	43	
	Cumulative Sensitivity Data		90.4%	374	338	87.4–93.4
**Metric**	**Reference**	**Method**	**Result**	**N**	**Detected**	**95% C.I.**
**Specificity**	Chera, 2019 [34]	Healthy donors, (*n* = 55) ^3^ banked blood, non-HPV related malignancy patients (*n* = 60)	97.4%	115	3	
	Routman, 2022 [36]	OPSCC tissue HPV negative by p16 and/or ISH	100%	7	0	
	Rettig, 2022 [37]	Academic center biobank samples, prospectively continuously curated, with no cancer or HPV related disease matched 10:1 with 10 cases of HPV-driven HNCs (*n* = 100) confirmed with p16 and/or ISH	100%	100	0	
	Cumulative Specificity Data		98.6%	222	3	97.1–100

^1^ p16 protein overexpression is a surrogate marker for HPV viral etiology in OPSCC and has a 5–10% false positive rate. False positive p16 tests may decrease the apparent sensitivity of NavDx. ^2^ Thirteen patients were p16 positive only with no ISH confirmation of HPV status. ^3^ The three detected individuals were: female (ages 20, 21, and 31) with low but detectable plasma. HPV16 DNA, no available history of HPV related disease, and are outside the intended use population for HPV-OPSCC surveillance.

**Table A3:** NavDx Positive and Negative Predictive Value for the Detection of Patients with Residual and/or Recurrent HPV-Driven OPSCC in the Post-Treatment Surveillance Population.

Metric	Reference	Method	Result	N	Detected	95% C.I.
**PPV**	Chera 2020 [35]	Biopsy proven recurrent HPV-driven OPSCC ^1^	100%	16	16	
	Berger 2022 [40]	Biopsy and/or imaging confirmed recurrent HPV-driven OPSCC ^2^	97.5%	80	78	
	Cumulative PPV Data		97.9%	96	94	95.1–100
	**Reference**	**Method**	**Result**	**N**	**Detected**	**95% C.I.**
**NPV**	Chera 2020 [35]	Imaging and clinical examination negative for recurrent OPSCC	100%	99	99	
	Berger 2022 [40]	Clinician reported status as “no evidence of disease” ^3^	95.4%	1256	1198	
	Cumulative NPV Data		95.7%	1355	1297	94.6–96.8

^1^ Prospective longitudinal study of NavDx in surveillance of 115 post-treatment patients with HPV-driven OPSCC. At the time of publication, 15 of the 16 patients with recurrence had been confirmed and the last patient was confirmed a few months thereafter. ^2^ Prospectively designed retrospective analysis of 1076 definitively treated HPV-driven OPSCC patients using NavDx in routine clinical care. NavDx was performed ≥ 3 months post therapy. At the time of publication 76 of the 80 patients with positive NavDx tests were confirmed to have residual/recurrent OPSCC. Two additional patients, thought to be NED at the time of testing, were documented to have recurrent OPSCC a few months post-publication. ^3^ Based on the clinician reported patient status at the time of testing, NPV was 95.4% on a per test basis, accounting for patients who had multiple negative tests.
